# Endoscopic endonasal resection of a clival chordoma with massive brainstem compression

**DOI:** 10.3171/2020.4.FocusVid.19942

**Published:** 2020-04-01

**Authors:** Mostafa Shahein, Thiago Albonette-Felicio, Giuliano Silveira-Bertazzo, Rafael Martinez-Perez, Marcus Zachariah, Ricardo L. Carrau, Daniel M. Prevedello

**Affiliations:** Departments of 1 Neurological Surgery and; 2 Otolaryngology–Head and Neck Surgery, The Ohio State University Wexner Medical Center, Columbus, Ohio; and; 3 Department of Neurosurgery, Aswan University Hospitals, Aswan University, Aswan, Egypt

**Keywords:** chordoma, skull base, endonasal, minimally invasive, surgical video

## Abstract

Chordomas are rare tumors that occur at an incidence rate of 0.8 per 100,000. Thirty-five percent of chordomas occur in the spheno-occipital region. We present a case of a clival chordoma that had severe brainstem compression. The patient had a 1-year history of slurred speech and left facial weakness (House-Brackmann 3). The endoscopic endonasal transclival approach gave a panoramic view of the region without the necessity of brain retraction or manipulation of the surrounding cranial nerves. Gross-total resection was achieved and no CSF leak was encountered postoperatively. The left facial weakness improved to House-Brackmann 1.

The video can be found here: https://youtu.be/DzW9Q6ckTHw.

## Transcript

Case presentation. This video demonstrates an endoscopic endonasal resection of a clival chordoma with a massive brainstem compression.

**0:27** Preoperative MRI. Patient was a 25-year-old gentleman who presented with a slurred speech, nystagmus, and mainly left facial palsy with a House-Brackmann 3 and some facial numbness as well.

**0:38** MRI showed a large tumor with impressive compression of the brainstem.

**0:43** Intraoperative preparation. Patient was brought to the operating room and positioned supine with the head turned to the right side and tilted to the left fixed in a Mayfield with pins with cranial nerve monitoring of 3rd, 6th, and 12th bilaterally. Face was also monitored for motor on the left side.

**1:02** Anterior and posterior ethmoidectomies. The endoscope was brought through the right nostril. Ethmoidectomies was performed initially through the right side anteriorly and posteriorly. A nasoseptal flaps was elevated on the right side, and we removed the bone of the posterior aspect of the septum and the mucosa of the left side was rotated anteriorly. A wide sphenoidectomy was performed, and initially we performed exposure of the dura of the sella with extension of the clivectomy inferiorly. Identifying and skeletonizing the carotid artery superiorly near the sella was essential for us to optimize the corridor drilling from internal carotid artery to internal carotid artery.

**1:07** Elevation of right nasoseptal flap.

**1:15** Posterior nasal septectomy.

**1:17** Reverse flap.

**1:21** Wide anterior sphenoidectomy.

**1:22** Drilling of the sella and clivus.

**1:32** Dissection of the tumor.

**1:45** We then used a 45° endoscope and removed the epidural component of the tumor, dissecting behind the pituitary gland as well as behind internal carotid arteries bilaterally. We spent time to optimize and to make sure all the components of the tumor extradurally was removed.

**2:03** We then used a 45° endoscope. We went behind the pituitary gland and removed the dorsum sellae and part of the posterior clinoid.

**2:12** Confirm the position of the basilar artery with the Doppler, and we followed the tumor to the intracranial component. We used two-hand, two-suction technique in order to dissect the tumor away from the brainstem very carefully, not to damage any of the neural tissue located intracranially.

**2:35** Forty-five-degree endoscope was essential to look around the dura. We opened the dura slightly larger than the defect caused by the tumor, and we were able then to go with a 45° all the way around to dissect the tumor from the basilar artery from the left side, as well as from the brainstem posteriorly and from the cranial nerves, particularly on the right side that we were able to visualize with the angled scope.

**3:05** Inspection of the intradural tumor cavity and surrounding cranial nerves. We saw cranial nerve V, VI, VII, and VIII on the right side as well as the sixth nerve on the left.

**3:12** We then finished the resection intradurally, and we moved ourselves to further dissection. We noticed there some residual in the tuberculum jugulare bilaterally, and after resecting the tumor completely we then performed reconstruction using a collagen matrix followed by fat graft followed by the nasoseptal flap that covered the entire skull base defect. We buttressed that in position using a NasoPore.

**3:27** Reconstruction of the skull base defect.

**3:47** Postoperative course. Patient did very well and was discharged the fifth day after surgery after improving the diabetes insipidus that was transient. The face completely went back to normal and discharged.

**3:57** Postoperative MRI. MRI showed a complete resection of the tumor and no signs of any complications. DWI was essential to prove that the tumor was completely resected since it was not very enhancing before surgery.
